# Using Multiscale Simulations as a Tool to Interpret Equatorial X-ray Fiber Diffraction Patterns from Skeletal Muscle

**DOI:** 10.3390/ijms24108474

**Published:** 2023-05-09

**Authors:** Momcilo Prodanovic, Yiwei Wang, Srboljub M. Mijailovich, Thomas Irving

**Affiliations:** 1 Institute for Information Technologies, University of Kragujevac, 34000 Kragujevac, Serbia; momcilo.prodanovic@gmail.com; 2 FilamenTech, Inc., Newton, MA 02458, USA; smijailo@gmail.com; 3 Department of Applied Mathematics, Illinois Institute of Technology, Chicago, IL 60616, USA; ywang487@iit.edu; 4 Department of Biology, Illinois Institute of Technology, Chicago, IL 60616, USA; 5 Department of Mathematics, University of California, Riverside, CA 92521, USA

**Keywords:** muscle, X-ray diffraction, multiscale modeling

## Abstract

Synchrotron small-angle X-ray diffraction is the method of choice for nm-scale structural studies of striated muscle under physiological conditions and on millisecond time scales. The lack of generally applicable computational tools for modeling X-ray diffraction patterns from intact muscles has been a significant barrier to exploiting the full potential of this technique. Here, we report a novel “forward problem” approach using the spatially explicit computational simulation platform MUSICO to predict equatorial small-angle X-ray diffraction patterns and the force output simultaneously from resting and isometrically contracting rat skeletal muscle that can be compared to experimental data. The simulation generates families of thick–thin filament repeating units, each with their individually predicted occupancies of different populations of active and inactive myosin heads that can be used to generate 2D-projected electron density models based on known Protein Data Bank structures. We show how, by adjusting only a few selected parameters, we can achieve a good correspondence between experimental and predicted X-ray intensities. The developments presented here demonstrate the feasibility of combining X-ray diffraction and spatially explicit modeling to form a powerful hypothesis-generating tool that can be used to motivate experiments that can reveal emergent properties of muscle.

## 1. Introduction

Synchrotron small-angle X-ray diffraction is the method of choice for nm-scale structural studies of striated muscle under physiological conditions [1,2]. In recent years, it has also come to the forefront as a technique for studying the relation of sarcomere structure to function in various myopathies using transgenic animal models and human biopsies [2]. While much can be done using existing interpretive frameworks [2], a critical barrier to progress has been that there are no generally applicable computational tools for modeling X-ray diffraction patterns from living muscles, limiting our ability to extract all the structural information potentially available. Conventional crystallographic approaches have not been fruitful because of the low resolution of the technique (typically 5–100 nm) and the cylindrical averaging, inherent in fiber diffraction patterns, that preclude conventional phasing techniques, such as multiple isomorphous replacement, that allow straightforward Fourier reconstruction of the electron densities [1,3]. New approaches are necessary to fully realize the potential of X-ray diffraction to provide unambiguous structural information from muscle.

### 1.1. Sarcomere Structure

**Figure 1:**
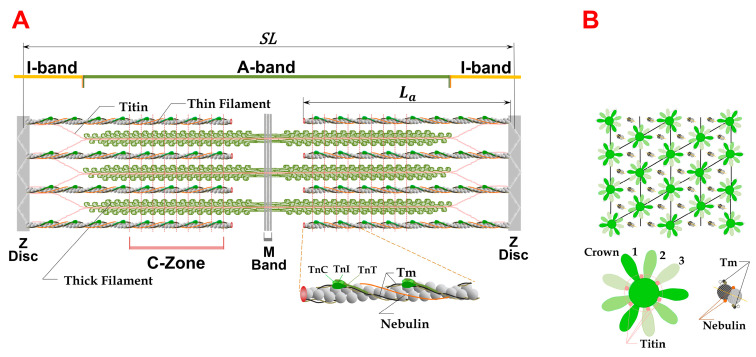
(**A**) Sarcomere lattice containing myosin containing thick and actin-containing thin filaments that overlap to varying degrees depending on the sarcomere length (SL). The A-band, I-bands, Z-disks, and M-bands are as indicated. Inset shows details of the thin filaments with the f-actin backbone with the regulatory proteins tropomyosin (Tm), the troponin complex (consisting of TnC, TnI, and TnT), and nebulin. Six titin molecules from each side of the M-line propagate along the surface of the thick filament and extend into the I-band to connect with the thin filaments near the Z-disks. Myosin-binding protein C (MyBP-C) is associated with every third crown in the C-zone (red dashed lines) and interacts with myosin near the S2-S1 junction or with actin in the thin filaments. (**B**) The hexagonal super lattice [1] showing the myosin crown orientations relative to the thin filaments. The azimuthal positions of six titin molecules associated with the thick filament backbone are indicated by pink circles. Myosin heads labeled 1, 2 and 3 are on three successive crowns. The thin filament detail shows the azimuthal positions of tropomyosin and nebulin as black and pink circles, respectively, on the surface of the actin filament.

Vertebrate skeletal muscle is composed of many multinucleated cells (fibers), ranging from about 20 to 200 µm in diameter. Within the muscle fibers are smaller cylindrical structures called myofibrils, about 1 to 2 µm in diameter, each comprised of many contractile units called sarcomeres connected end to end (Figure 1). Individual sarcomeres consist of a hexagonally packed lattice of myosin containing thick filaments with inter-digitating actin-containing thin filaments. The ends of one sarcomere and the beginning of the next are marked by the Z-disc. The thin filaments are attached at one end to a Z-disc and extend toward the center of the sarcomere. The region of the sarcomere where thick and thin filaments overlap is called the A-band, and the region where thin filaments do not overlap with thick filaments close to the Z-disc is called the I-Band. In the center of the sarcomere, thick filaments are connected via a structure called the M-band. The region on either side of the M-band where thin filaments do not overlap with the thick filaments is called the H-zone.

Thick filaments are primarily composed of the motor protein myosin II along with a number of accessory proteins, including myosin-binding protein C (MyBP-C), and are also closely associated with the elastic protein titin where each half myosin filament is associated with six titin molecules [4,5,6,7]. Along the length of the thick filament groups of three myosin heads form crowns axially shifted by 14.3 nm and azimuthally rotated by 40°. Myosin-binding protein C appears in groups of three along the thick filaments associated with every third crown in the C-zone (red dashed lines) and interacts with myosin near the S2-S1 junction or with actin on thin filaments.

The thin filaments consist primarily of actin, along with the accessory protein nebulin, which is believed to act as a molecular ruler specifying filament lengths among other functions [8,9,10]. Thin filaments also contain the regulatory proteins tropomyosin and the troponin complex (consisting of troponin C, troponin I, and troponin T), along with a number of other minor components. In the 3D sarcomere lattice, each myosin filament is surrounded by six actin filaments and each actin filament is associated with three myosin filaments.

### 1.2. The Equatorial X-ray Diffraction Pattern from Striated Muscle

**Figure 2:**
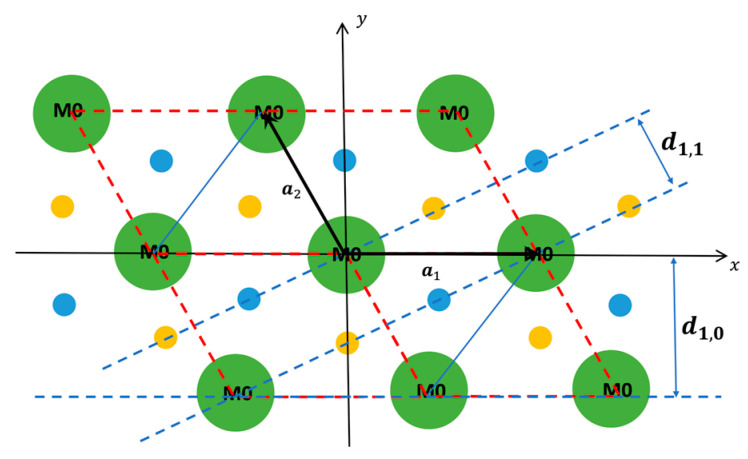
Arrangement of thick and thin filaments in the sarcomere lattice. Large green circles are myosin containing thick filaments. Yellow and blue circles are symmetry-related actin containing thin filaments. The red dotted lines indicate crystallographic unit cells. The *1,0* and *1,1* lattice planes are indicated as blue dotted lines. The lattice vectors **a_1_** and **a_2_** are indicated by black arrows.

The strongest features in X-ray fiber diffraction patterns of striated muscle are the innermost 1.0 and 1.1 equatorial reflections arising from the densities of the thick filaments and thin plus thick filaments, respectively [1,11]. With only these two reflections, one can accurately determine the inter-filament lattice spacing, d_1,0_, proportional to the distance between thick and thin filaments (i.e., (2/3) d_1,0_) (Figure 2), as well as the I_1,1_/I_1,0_ equatorial intensity ratio. Low values of I_1,1_/I_1,0_ indicate that the myosin heads are primarily associated with the thick filament backbone, whereas high values indicating that the myosin heads have moved outward to be more associated with thin filaments [2,11]. Both d_1,0_ and I_1,1_/I_1,0_ are important physiological parameters that are hard or impossible to obtain any other way from muscle fibers under hydrated, physiological conditions.

It has been shown that I_1,1_/I_1,0_ is strongly correlated with force in active rodent cardiac muscle [12,13] and mammalian skeletal muscle [14,15] so that *I_1,1_/I_1,0_*, under these conditions, can be used to estimate the number of cross-bridges attached to actin during contraction. *I_1,1_ /I_1,0_* can also be used to estimate the relative degree of association of myosin heads with thin filaments under resting conditions and has been widely used to characterize myocardium from transgenic mouse disease models [2]. Under resting conditions, *I_1,1_/I_1,0_* has also been used to study the radial movement of myosin heads in the presence of various compounds [14,16,17] and in the presence and absence of phosphorylation of various sarcomeric proteins [18,19,20]. We now have a new appreciation that the radial movements of heads resulting in higher *I_11_/I_10_* values are primarily due to transitions from heads in quasi-helically ordered OFF states (unable to interact with actin), held close to the thick filament backbone at low radius to disordered ON heads at a higher radius (able to interact with actin). These transitions are widely assumed to correspond to transitions from the energy-sparing super-relaxed state (SRX) to one or more disordered relaxed (DRX) state(s), although this strict relationship has recently been called into question [21]. In this paper, we will use a more generic term, the “Parked State” (PS), defined as heads located close to the thick filament actin (unable to interact with actin) [22], which is agnostic as to whether heads are helically ordered or in the SRX.

While X-ray diffraction studies aimed at determining only d_1,0_ and I_1,1_/I_1,0_ have been very useful in the past and continue to be useful to date, early computational simulation studies [23,24] showed that there are multiple factors that can influence I_1,1_/_1,0_, making unambiguous interpretation difficult under some conditions. While changes in I_1,1_/I_1,0_ are usually interpreted as changes in the radial positions of myosin heads, there are other factors that can affect *I_1,1_/I_1,0_* [24]. One such factor is that the flexibility of thin filaments in resting muscle may cause them to be poorly localized around their lattice positions, leading to low values of *I_1,1_/I_1,0_* [23,25]. Thus, to unambiguously interpret changes in *I_1,1_/I_1,0_* as being due to radial movements of myosin heads and/or cross-bridge binding to actin, one needs other corroborating evidence, such as the changes in the position of layer line peak intensities or evidence from physiological data such as stiffness. In the absence of such data, *I_1,1_/I_1,0_* is still a useful operational metric to characterize a particular muscle system such as relaxed, skinned myocardium from a transgenic mouse disease model [2]. An additional complexity is that changes in I_1,0_ and I_1,1_ have been shown to follow different time courses in a number of different muscle systems [3,26,27,28]. Modeling studies by Eakins et al. of the time courses of the *1,0* and *1,1* reflection intensities in contracting bony fish muscle suggested that the relative intensity changes of the *1,0* reflections, on their own, appeared to be a better measure of the number of attached force-producing heads than the *I_1,1_/I_1,0_* ratio, observed as a decrease in *I_1,0_* as force increases. Consistent with this notion, experiments of Reconditi and colleagues [28] on contracting frog skeletal muscle showed a linear relationship between *1,0* intensity and tension, whereas in bony fish muscle, I_1,1_ appears to correlate with weakly bound myosin heads [26]. Given these anomalies, a more rigorous approach, such as described here, for predicting the equatorial reflections is needed to unambiguously interpret the diffraction data, motivating the current studies.

### 1.3. Using Multiscale Simulations to Predict X-ray Fiber Diffraction Patterns from Muscle

**Figure 3:**
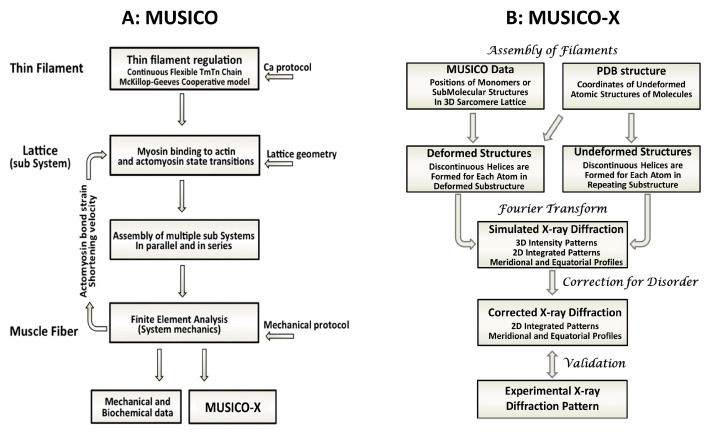
Overview of MUSICO and relation to MUSICO-X. (**A**) MUSICO fiber simulations provide mechanical data at the level of muscle fiber and biochemical and structural data at molecular level within sarcomere. (**B**) MUSICO-X predicts X-ray diffraction patterns from structural data obtained from MUSICO fiber simulations and available PDB structures. These simulations provide X-ray patterns from the structural data using Fourier transform that alter correction for the disorders and can be compared and validated by observed X-ray patterns.

Here, we report a novel “forward problem” approach using a spatially explicit multi-scale simulation package to simultaneously calculate the equatorial small-angle X-ray diffraction pattern and the force output from striated muscle and compared to experimental force and diffraction data. The MUSICO (Muscle Simulation Code) simulation platform was developed initially to predict only physiological (functional) data [29]. The 3D, spatially explicit, multi-sarcomere structures embodied by the MUSICO simulations are based on the best structural and functional data available so that the positions of all sarcomere components at any instant in time are known. We realized that if these individual sarcomeric components were to be provided with realistic electron densities, it should be possible to predict small-angle X-ray diffraction patterns by straightforward Fourier transformation. We first tested this concept by extending MUSICO to predict X-ray fiber diffraction patterns of extensible thin filaments [30]. Emboldened by the success of this development, we undertook a long-term project to extend MUSICO with a new computational tool, “MUSICO-X”, to predict the X-ray fiber diffraction patterns from muscle with the goal of developing a hypothesis-generating and -testing tool in which the best available mechanistic knowledge is tested against the best available mechanical and X-ray diffraction data. The overall scheme of MUSICO and MUSICO-X is given in Figure 3.

The first step toward this goal was to build a 2D-projected electron density model for the sarcomere using known Protein Data Bank (pdb) structures of the individual sarcomere components. By using MUSICO, we were able to generate families of thick–thin filament repeating units, mimicking the distributions in muscle fibers, each with their individually predicted occupancies of different populations of myosin heads including inactive heads in an ordered, “parked state” close to the thick filament backbone, disordered unattached heads, and active heads bound to actin [22]. This approach enabled us to construct electron density distributions, which can be used to generate predicted equatorial X-ray reflection intensities that can be compared to experimental values. Moreover, we show how, by adjusting only a few selected parameters, we can achieve a good correspondence between experimental and predicted X-ray intensities. It is important to note that this process is very different from the usual approaches that attempt to fit a structural model to a set of data. Here, we are trying to predict functional and structural properties of muscle from a spatially explicit 3D simulation derived, as much as possible, from first principles and available structural and functional information. The end goal is, rather than to refine a structural model, to adapt the simulations so that they can predict the spatial distributions of bound crossbridge states and the populations of myosin heads in the parked state using the X-ray data and the experimental tension as constraints. This first demonstration of a successful prediction of experimental X-ray diffraction intensities from a spatially explicit simulation provides a stepping stone toward prediction of the entire 2D X-ray fiber diffraction of muscle. Even at the current stage of development, the ability to simulate just the equatorial pattern will provide a potent interpretive and hypothesis-generating tool for realizing the full potential of X-ray diffraction, in concert with the mechanical data, to reveal the structural dynamics underlying muscle contraction.

## 2. Results

### 2.1. Estimation of Number of Bound Myosin Heads during Isometric Contraction Using MUSICO

In this study, we used MUSICO simulations (see Methods) to estimate the number of active myosin heads bound to actin and heads sequestered in an inactive parked state closely associated with the thick filament backbone in a quasi-helically ordered configuration [31]. Experimental force traces were obtained from six EDL tetanic rat muscles undergoing maximum isometric tension. To simulate the average tetanic forces from these experiments with MUSICO, we used as input parameters the experimental average sarcomere lengths (*SL*), interfilament spacings (*d*_10_), and corresponding spacing between actin and myosin (*d_AM_*) (Table 1). The length of the actin filaments (*L_a_*) assessed using fluorescently labeled tropomodulin was 1.16 ± 0.04 μm for rat EDL [32]; thus, for MUSICO simulations, *L_a_* was set to 1.15 mm for EDL muscle assuming 31 and 30 TnC units, respectively, on each actin strand of the actin double helix, axially separated by 38.3 nm.

While it is often assumed that 100% of the heads in resting muscle are helically ordered, here considered to be in the parked state, there are indications that this is not the case in rodent skeletal muscle. Ma et al., 2018 [14], showed that when resting mouse EDL muscle was treated by blebbistatin, the fourth myosin layer line, MLL4, increased in intensity by 80% over its resting value. If one assumes that blebbistatin treatment sequestered all of the heads into the parked state, this implies that the percentage of heads in resting EDL muscle is –75% under the assumption that layer line intensity is proportional to the square of the number of diffracting units in these helically ordered myosin heads. Under isometric contracting conditions, the layer lines do not completely disappear when a residual layer line intensity is –30% of its value at rest, implying that about 50% of the resting head population remains in the PS during isometric contraction. These 50% remaining heads of the 75% ordered heads of the total at rest implies that –35% of the total number of heads remains helically ordered under isometric contraction conditions. We used these estimates from mouse EDL muscle to provide the initial upper and lower boundaries for parked-state head populations for rat EDL skeletal muscle in the simulations.

To match the number of myosin heads in the PS in relaxed and contracting muscles, the amplitude of the [Ca^2+^] dependent forward rate from PS to MDPi state, $k_{PS}^{max}$, was lowered 1.8 times, while the baseline rate $k_{PS}^{0}$ was increased 8–10 times from the values used in previous cardiac muscle twitch simulations [33]. Using these new values for $k_{PS}^{max}\;$ and $k_{PS}^{0}$ in MUSICO simulations resulted in best estimates for the fractions of myosin heads in the PS which is ~80 % in resting EDL muscle, and ~34% in contracting EDL muscle. Finally, to match the observed tension time courses in fast EDL skeletal muscles, two state transition rates in the actin–myosin cycle had to be adjusted, namely, the myosin binding rate, $k_{A}$, and the ADP release rate, $k_{D}$ (Table 2), while other parameters in the crossbridge cycle are taken from [33]. A full list of parameters used in the simulations is given in (Table S2).

Predicted force traces for EDL muscles using MUSICO closely matched the experimental observations of tension development, as shown in Table 3. The predicted fractions of unbound and bound crossbridges, as well as the fraction of crossbridges in the parked state (PS) in both contracting and relaxed muscle, are shown in Table 4.

### 2.2. Comparison of Experimental and Simulated Equatorial X-ray Reflection Intensities

Table 5 compares experimentally measured and simulated equatorial X-ray diffraction reflection intensities from rat extensor digitorum longus (EDL) muscle. Experimental measurements are the averages of the six individual measurements from EDL muscles used for the force measurements. Intensities have been normalized to that of the 1.0 equatorial reflection [23] so that the intensities are represented as a percent of the 1.0 intensity. Simulated intensities (also normalized to the 1.0 reflection) presented as the mean and standard error of the mean (Error), are the average of the predicted intensities from 10 individual unit cells, each consisting of one thick filament and two thin filaments (Figure 2), with myosin head distributions calculated by MUSICO and electron densities calculated as described above.

### 2.3. Refinement of Thick Filament Backbone Parameters for the Thick Filament Electron Density Model

**Figure 4:**
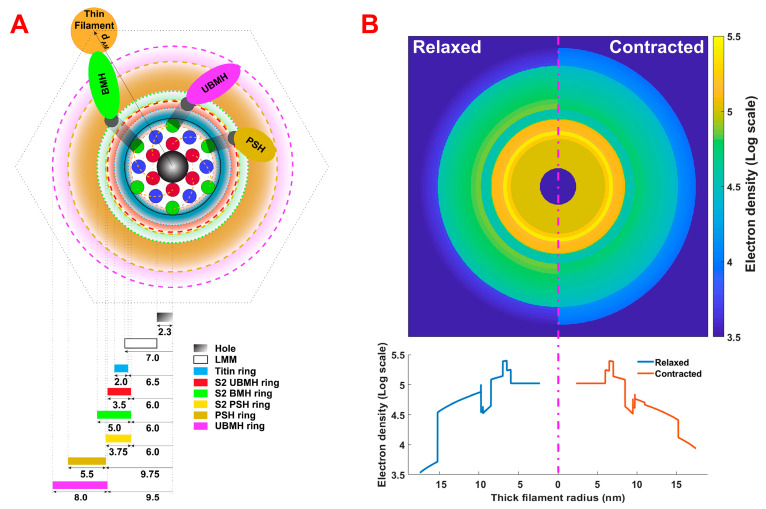
(**A**) Structural arrangement of thick filament including hexagonal packing of 18 LMMs in three layers in the backbone, titin, and S2s. Myosin heads are assigned to either the ordered parked state (PSH), the disordered unbound state (UBMH), or the actin-bound state (BMH). Colored bars represent specific rings of density around the backbone. Blue and red dots in the thick filament backbone region represent LMM “ribbon motif’s” belonging to two different layers. Dashed lines represent boundaries of specific rings. The distance between thin and thick filament centers is denoted with *d_AM_*. (**B**) Thick filament electron density distributions in relaxed and contracted muscles. The number of crossbridges in each state is taken from MUSICO simulations. The 2D density distributions are shown in the upper panel. The lower panel represents the projection of electron densities along the thick filament radius.

In the thick filament electron density scheme (Figure 4), the outer radius for the S2s attached to the parked-state heads and the inner and outer radii of the ring containing the parked-state heads are not well-defined because of the lack of information concerning the degree of tilt of the parked-state heads away from projection plane. We adjusted these values to agree with the relaxed equatorial intensity data using a brute-force fitting approach of the two key parameters defining the radial center of mass of the PS heads and the thickness of the PS region. During the fitting procedure, the parked-state fraction of all crossbridges was kept fixed, as provided by MUSICO simulations (Table 4), and the inner radius of the unbound myosin heads was set to 9.5 nm. Table S1 indicates the sensitivity of the fits to these two parameters. The best-fit parameters to the experimental data for EDL muscle are shown in Table 5. We found that the results within the physically realistic range (12.5 ± 1 nm for the radial center of mass of the PS heads and 4–6 nm for the thickness of the PS region) were not very sensitive to the exact values for these parameters (Table S1 and Figure S4). Thus, we chose the outer radius of S2 attached to PS heads to be 9.75 nm and the inner and outer radii of the myosin heads in the PS to be 9.75 and 15.25 nm, respectively, as the best compromise for subsequent calculations.

### 2.4. Simulation of Equatorial Diffraction Pattern from Contracting Muscle

Having established the best-fit parameter values for the thick filament backbone structure using the equatorial data from resting muscle and the values for the population of heads (attached, unattached, and in the PS) from Table 4, we simulated the equatorial diffraction patterns from contracting muscle with the results shown in Table 5. The only parameters adjusted for these fits were the two temperature factor terms $\Delta_{M}\;and\;\Delta_{A}$ for the thick and thin filaments, respectively, to account for the different flexibilities of the thick and thin filaments [23] that affect the degree of localization of the filaments to their lattice positions. Assuming only one temperature factor term gave similar fits but with somewhat greater R-factors. The predicted phases of the reflections were always the same, namely “++−−++−−”where “+” indicates a phase angle of 0 radians and “—” indicates a phase angle of 𝜋 radians. Parameters were adjusted by calculating intensities for all possible values of $\Delta_{M}\;and\;\Delta_{A}$ on a fine grid and selecting values that yielded the lowest R factor. It is important to note that, using this approach, there is no problem with being trapped in local minimums in the R-factor distribution since all reasonable values of $\Delta_{M}\;and\;\Delta_{A}$ are being interrogated. Since the eight observed X-ray reflection intensities were normalized to that of the 1.0 reflection, there were effectively seven observables and two free parameters in the fits, so there was no risk of overdetermination in these fits.

## 3. Discussion

### 3.1. Overview

Here we show that a physically realistic model based on currently available structural data for the myofilament densities combined with predictions of myosin head distributions from multi-scale simulations can predict X-ray diffraction intensities that provide close matches to those determined experimentally from resting and contracting intact rat EDL muscle (Table 5). This model differs in important ways from previous efforts to simulate the equatorial pattern. These earlier efforts using crude cylinder models [23,24,34,35,36] had multiple parameters, usually the same or greater than the number of observable X-ray reflections, so they ran the risk of overdetermined solutions. They also made no use of the force data. Thick filament backbones were previously modeled as one uniform cylinder containing all of the mass of LMM, titin, myosin, and S2, whereas in our new model these are assigned to their own separate regions. Masses of bound myosin heads, typically modeled as a cylindrical shell of density centered on thin filament, were determined by fitting the data as a free parameter. While the unbound heads could be confined to a particular radius from the center of the backbone, the possibility of an ordered “parked state” was not considered. In contrast, our “forward problem approach” uses known structural information, starting with PDB structures, when available, with a much more detailed thick filament structural model than used in previous studies. While our thick filament backbone model involved some poorly defined parameters, in the initial fitting of the resting equatorial data, the values of just two of these parameters needed to be adjusted within a narrow range of physically realistic values to provide reasonable matches of predicted and experimental intensity. Remarkably, once best-fit estimates for these thick filament backbone parameters were obtained using the resting muscle data, it was possible to achieve a good match to the contracting equatorial diffraction data with the experimental data by only adjusting the Gaussian temperature factors to minimize the R-factor when the numbers of bound myosin heads associated with the thin filament and parked state myosin heads associated with the thick filament were estimated by the MUSICO simulations using the experimentally observed experimental tension values. By combining these different sources of information, this approach avoids the risk of overdetermined solutions, so the results are much more likely to be meaningful and useful.

### 3.2. Significance of Study

The goal of this development was to expand the scope of a spatially explicit simulation package to predict the structure and function of striated muscle as a tool for providing insight into the multiple processes inside the muscle that contribute to the macroscopic observables that can only report the net result of such processes acting together. Our new equatorial modeling component of MUSICO-X represents a first step in incorporating structural data, in addition to the mechanical data as constraints to the simulation. By incorporating prior structural information and matching the predicted force to the observed force, the distributions of attached, unattached, and parked-state myosin heads can be used to construct electron densities that are Fourier transformed to generate predicted diffraction intensities that can be compared to the experimental diffraction patterns. By determining what adjustments to the model are required to match the X-ray intensities, the electron density distributions are refined. In this way, the interaction between MUSICO-X and the X-ray diffraction data is a “two-way street” where simulations iteratively improve as new information comes available, while the predictions at each intermediate stage of development will provide guidance as to what might be the most valuable experiments to do next. While the way the MUSICO simulation platform is constructed facilitated these studies, this general approach could be applied to other spatially explicit computational models of muscle contraction.

### 3.3. Future Directions

Being able to simulate both relaxed and contracting X-ray diffraction patterns of at least one muscle system provides vindication for our overall approach combining multi-scale simulations and X-ray fiber diffraction. This is only the beginning, however. The matches obtained between prediction and experiment, while close, are not perfect indicating that our models for the electron densities are not perfect and can benefit from further refinement. Our model for the backbone structure is a hypothetical structure based on the best information currently available. It should soon be possible to replace these hypothetical structural models using information from real high-resolution structures determined by cryo-electron microscopy. Such structures exist for thin filaments [37,38,39,40] and thick filaments in tomograms [10,41]. At this time`, high-resolution cryo-electron microscopy structures of myosin thick filaments are only available from insect model systems [42,43,44], but we may expect structures of vertebrate thick filaments soon. These new structures will allow us to replace hypothetical structural models with real density distributions from these structures in our simulations. It should be emphasized that cryo-EM structures are necessarily static structures taken under a given set of conditions, so the simulations will provide a mechanism to predict the ways these structures change different static and dynamic conditions.

Our ultimate goal is to predict the entire 2D diffraction pattern from muscle, including all the actin and myosin meridional reflections and the layer lines. We have made a start at this process by first establishing a methodology [30] for predicting the 2D diffraction pattern from non-uniformly strained actin-based thin filaments (non-regulated). To predict the diffraction pattern from regulated thin filaments, troponin, tropomyosin, and nebulin (if applicable) will need to be added to the structure, incorporating structural details from current thin filament cryo-EM structures as appropriate. The next major task will be to predict the meridional and layer line reflections from the thick filaments by extending our methodologies for predicting the 2D diffraction patterns from non-uniformly strained thin filaments to thick filaments. We also plan to extend our approaches to biomedically relevant systems such as cardiac muscle. To do this, however, we need to have adequately characterized structural information such as thin filament length distributions, thick filament backbone structure, the radial distribution of densities caused by myosin-binding protein C, among other poorly known characteristics.

While there is still much work to do, the developments presented here demonstrate the feasibility of combining X-ray diffraction and spatially explicit modeling to form a powerful hypothesis-generating tool that can be used to motivate experiments that can be used to reveal emergent properties of muscle. Model predictions can be improved by incorporating new structural information and implementing new concepts and mechanisms revealed by experiments. Using this approach, the model and its ability to make predictions will evolve to provide an ever-more realistic representation of muscle behavior in health and disease.

## 4. Materials and Methods

### 4.1. Simulation of Myosin Head Distributions from Mechanical Data

The spatially explicit simulation platform MUSICO [29] integrates the action of molecular (crossbridge) forces into a 3D sarcomere structure and defines the configurations of thin and thin filaments at any instant of time. Its modular structure consists of four major parts: (1) a detailed multi-sarcomere geometry for the simulation of myofibril contraction [7,29], (2) a series elastic element (SE) for accounting for the changes in sarcomere length when the muscle is held at a fixed length [22], (3) a six-state crossbridge cycle that includes a “parked state” [22,33,45], and (4) a model for thin filament regulation defined by a six-state scheme for one or two Ca^2+^ ions binding to troponin (Tn) coupled with a continuous flexible chain (tropomyosin) model used for regulation of myosin binding to thin filament(s) [46,47,48].These elements comprise a unified description of muscle function using (1) the kinetics of the underlying biochemical reactions to define a state rate transition matrix that integrates the biochemical reactions of the actomyosin cycle and the kinetics of Ca^2+^ regulation at the molecular scale and (2) a finite element model in which all geometrical factors, and the constitutive elastic properties of filaments and crossbridges, are assembled in the system’s stiffness matrix at the myofibrillar scale. All molecular transient kinetic transitions are defined as Monte Carlo processes and are integrated with the iterative solution of the equilibrium equation using nonlinear finite element analysis [29]. Simulations are performed for prescribed boundary conditions (external forces or displacements) and activation by [Ca^2+^] transients [22]. The simulations typically include multiple sarcomeres where each half sarcomere lattice consists of 500 half-thick filaments and 1000 thin filaments. Each half-thick filament consists of ~150 myosin molecules projecting from each side of the M-line. Due to the stochastic nature of the Monte Carlo method used in MUSICO simulations, each thick filament and each thin filament has a potentially different spatial distribution of bound crossbridges, unbound myosin heads, and myosin heads in the “parked state”. What arises from the simulation is a spatially explicit position and biochemical state for each myosin, actin, and regulatory protein at any time point in an experimental protocol. By adding the contribution of each kinetic reaction individually, weighted by its probability, this ensemble can be used to predict the force and length output, which can be compared to the observed mechanical data.

The simulations that match the observed tensions generate families of thick–thin filaments repeating structural units with differing numbers and spatial positions of myosin heads bound to actin and unbound myosin heads distributed between the active states (able to interact with actin) and parked states (unable to interact with actin). Each of these different thick–thin filament repeating units can be individually assigned electron densities and Fourier transformed to generate the predicted diffracted intensities from that repeating unit. These predicted diffraction patterns may then be summed to produce the expected diffraction pattern from the half-sarcomere structure used in the simulation. Since the position of every molecule is defined in the explicit 3D sarcomere model, the simulation can be defined by relatively few parameters that are, to the extent possible, fixed so that all adjustable parameters have physical meaning with a strictly defined range of parameters and their number minimized to prevent overdetermination of the fits.

### 4.2. Electron Densities

To convert the molecular positions resulting from the MUSICO simulations to projected electron density distributions, we used Protein Data Bank (PDB) structures for individual sarcomere components, when they were available, to estimate the electron densities in each region or subunit of the thick and thin filaments or molecular structures in their surroundings. This was done by extracting information concerning the type, position, number of electrons in each shell, and the van der Waals radius for each atom in the PDB file. With this information, it is possible to create spherical electron density distributions, each with a mass that equals the number of electrons in the shell for each atom and then calculate the volume projection along the Z (longitudinal) axis. Finally, the projected 2D electron density for each atom is placed into a 2D matrix representing the projected density onto a plane perpendicular to the long axis of the sarcomere, according to its X and Y coordinate positions, and added to the previously calculated electron densities in the matrix to build up the two-dimensional electron density map.

#### 4.2.1. Thick Filament Backbone Densities

A difficulty for calculating thick filament backbone densities is that there are currently no high-resolution structural models for the vertebrate thick filament backbone structure. Recently, a high-resolution cryo-EM structure for the *Lethocerus* flight muscle thick filament was published [42] showing so-called ”ribbon motifs” for the packing of the LMM portions of myosin. We were able to assemble a model for the electron density distribution in the thick filament backbone by incorporating the ribbon motifs into the Squire 1973 model for the three-stranded myosin thick filament backbone for vertebrate muscle [49,50] as modified by [51]. The outer radius of the backbone is 7 nm, allowing the packing of 18 LMMs into three hexagonal layers rotated relative to each other by 30° (Figure 4). The average number of LLMs in cross-section (18) was calculated from the length of LLM and the crown periodicities of 14.3 nm [51]. This newly constructed backbone structure has a hole in the middle with a radius of 2.3 nm. The number of LMMs varies from 3 at the tip of the filament to 18 in the long middle section, increasing further in the bare zone region where LLMs from the opposite sides of the thick filament overlap with a radius of 7.9 nm [52]. The density of the thick filament backbone was calculated using the high-resolution structure for human β-myosin subfragment S2 (2FXM.pdb) [53,54] as a surrogate for LMM for which high-resolution structures are not available. The density of the myosin backbone is calculated as the ratio of LMM length and S2 subfragment length, multiplied with the electron density of the S2 subfragment, with the total number of LMM portions of the myosin tails taken to be 18 (Figure 4).

#### 4.2.2. Myosin S2 Region Densities

The myosin subfragment 2 (S2) region densities were created by adding together the electron densities of three distinct rings containing, respectively, S2s attached to myosin heads in the PS, S2s attached to unbound myosin heads, and S2s attached to myosin heads bound to the actin filament. The relative populations of S2s in each of these categories will be expected to change when muscle is activated. The total electron density of each part of the S2 density distribution is calculated by multiplying the number of crossbridges contributing to that component of the S2 region, taken from the MUSICO simulations, with the ratio of the S2 length and the length of the S2 subfragment multiplied by the S2 subfragment density. We then smear the total electron density of each part of S2 around the corresponding ring. Each density ring has a trapezoidal shape in the radial direction of the cross-sectional density profile, so there is higher density closer to the LMM core that then decays proportional to the radius (Figure 4). The S2 density rings start at 6 nm from the center of the backbone and spread out up to 9.75 nm for myosin heads in the PS. The S2 densities for the unbound myosin heads extend out to 9.5 nm, whereas for the actin-bound myosin heads, they extend outward to 11 nm (Figure 4). Because of the variations in the spatial position of the myosin-head–S2 junction, these radii represent estimated average values.

#### 4.2.3. Titin Densities and Densities of Unbound Myosin Heads Centered on the Thick Filament

It is known that six titin molecules surround each thick filament backbone, but their exact positions are not defined. For our purposes, the titin contribution to the electron density was modeled as a ring around the thick filament backbone with an inner radius of 6.5 nm (slightly inside the myosin filament backbone) and an outer radius of 8.5 nm. These radii are similar to those reported in Squire et al., 1998 [50]. The electron density of titin was then calculated by taking the portion of molecular weight (MW) of six titin molecules that are associated with the A-band. The molecular weight of the portion of each titin molecule in the A-band is conserved across different skeletal (fast and slow) and cardiac muscles, having value of ≅2 MDa [4].

The number of crossbridges in the “parked state” (PS) closely associated with the thick filament backbone, as defined above, is estimated by the MUSICO simulations, and the remaining number of unbound (disordered) crossbridges is calculated by subtracting the number of bound crossbridges plus the number of crossbridges in the PS from the total number of crossbridges. Each crossbridge consists of two myosin heads. The electron densities of heads in the PS or in the disordered unbound state are calculated by multiplying the density of one myosin head with the number of heads, i.e., two times the number of crossbridges in the corresponding state. Alternatively, we have enabled an option to specify the ratio between the ordered unbound myosin heads in the PS and the disordered unbound heads, while keeping the number of bound heads as predicted by MUSICO simulations. The density of PS heads is represented in our model as a 5.5 nm thick ring surrounding the backbone (PSH ring in Figure 4). The width of a myosin head is about 5.5 nm, and heads in the PS are assumed to lie on the backbone surface. Consistent with the experimental observations [14], which show that the center of mass of helically ordered heads, presumably in the PS, lies 12.5 nm from the center of the backbone, the inner radius of myosin heads in the PS was set to 9.75 nm, while the outer radius is 15.25 nm. The electron densities of the remainder of the unbound myosin heads, as well as the second heads corresponding to the heads bound to actin, are shown in a separate ring (UBMH ring in Figure 4). The outer radius of the unbound head distribution is set to 17.5 nm, i.e., the maximum radius observed of the three-stranded vertebrate myosin (thick) filament [55]. Because the unbound myosin heads are tilted toward the muscle fiber axis, the projection of the longer side of the myosin head in the projection plane is shorter and is approximately on average 8 nm in the radial direction. The inner radius of the unbound myosin heads is calculated from the outer radius (17.5 nm) minus the head radial projection (8 nm) so that the calculated inner radius becomes 9.5 nm (Figure 4). Both the PSH ring and the UBMH ring densities also have trapezoidal cross-sectional profiles reflecting the reduction in density with increases in radial distance from the center of the thick filament.

#### 4.2.4. Thin Filament Densities

Electron densities were estimated starting with a PDB structure, 6KN8, for a portion of the thin filament that includes 15 actin monomers with tropomyosin and troponin, and then repeating this motif along the filament. The thin filament electron density is obtained by summing up the electron density of the 6KN8 structure, which rotates for 27.7° after every 14th monomer up to the end of the thick filament, or to the last actin site where the myosin head is attached. PDB structures for nebulin do not currently exist; thus, to incorporate nebulin we used, as a surrogate, the PDB data for tropomyosin but rotated it by 90° to be azimuthally displaced with respect to the position of tropomyosin units [10]. Note that the thin filaments in the I-band are generally assumed to be too disordered to contribute to the X-ray pattern from the hexagonally packed myofilaments in the A-band [1], so only densities within the A-band are considered in our calculations.

#### 4.2.5. Densities for Pre- and Post-Powerstroke Myosin Heads

**Figure 5:**
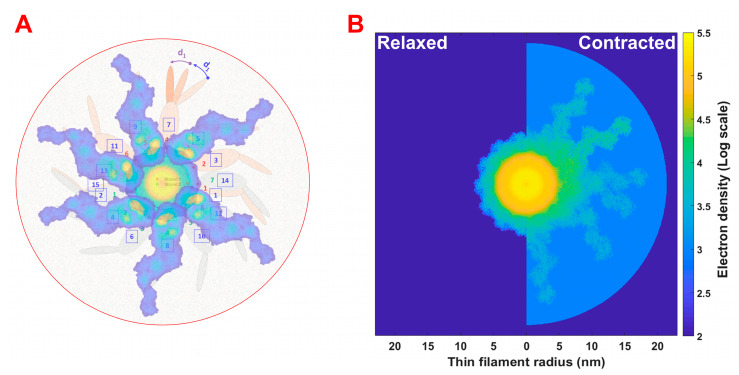
(**A**) Structural representation of thin filament with attached crossbridges in pre- and post-stroke states (denoted as d_1_ and d_2_). The numbers in squares represent monomers packing along an actin double-stranded helix and possible positions where myosin heads can attach. Strand 1 is labeled with red and Strand 2 with green symbols and numbers. (**B**) Electron densities of the thin filament in relaxed and contracted muscle. Thin filament density includes actin monomers, tropomyosin, troponin, and nebulin in the A-band. In contracting muscle, there are myosin heads in pre- and post-stroke states bound to actin along with a uniform cloud of non-bound second heads associated with attached heads.

Electron densities of myosin heads were created using PDB structures myo5_lev.pdb (reported in Holmes et al. 2004 [56]) for a post-powerstroke structure and 1QVI.pdb for a pre-powerstroke structure. Using calculated azimuthal angles and lever arm positions (post- or pre-powerstroke state) from the spatial positions of myosin heads in the 3D MUSICO simulations, electron densities for each attached myosin head were added around the thin filament densities. Densities of non-bound myosin heads, which form pairs with the bound myosin heads, were added as a uniform cloud of electron density centered on the thin filament (Figure 5).

### 4.3. Calculation of Predicted Equatorial Diffraction Pattern from the Electron Densities

In muscle cells, the thick and thin filaments form a two-dimensional hexagonal lattice, in which the thick filaments are located at the lattice points and the thin filaments are located at the trigonal positions (between three thick filaments), as shown in Figure 2. The crystallographic unit cells, indicated as red parallelograms, each contain the equivalent of one thick (since the thick filaments are shared between parallelograms, each parallelogram contains two 1/3 segments of a thick filament, plus two 1/6 segments of a thick filament) and two thin filaments within its boundaries. This lattice produces a characteristic set of equatorial reflections. According to diffraction theory [1,24], the equatorial reflections arise from the density of the myofilaments in the A-band of the sarcomere projected onto a plane perpendicular to the long axis of the muscle fiber. One can draw imaginary planes through the crystallographic unit cell corresponding to various values of the crystallographic Miller indices, (*h,k*), where *h* and *k* are integers. The two strongest pairs of X-ray reflections in the X-ray diffraction patterns from vertebrate striated muscle are the *(1,0)* and *(1,1)* reflections with intensities *I_1,0_* and *I_1,1_*, respectively. In addition, weaker, so-called higher-order, equatorial reflections can often be observed. The positions of these peaks are determined by the selection rule for a hexagonal lattice *S_h,k_ = S_1,0_*√*(h^2^ + k^2^ + hk*), where *S_1,0_* is the distance from the center of the X-ray pattern (incident beam position) to the *1,0* reflection. Any equatorial reflection arises only from those parts of the sarcomere where the angle between the unit cell of the filament lattice and the beam is equal to the Bragg angle for this particular reflection. Since the orientation of the filament lattice in individual myofibrils is random with respect to the incident X-ray beam, the diffraction pattern of a muscle is the azimuthal average of the square of the Fourier transforms of the electron density distribution. Each myofibril, therefore, acts as a single crystallite contributing intensity to only those reflections that are at the appropriate Bragg angle to the incident beam. Because even a single muscle fiber consists of thousands of myofibrils, each at random angles with respect to their neighbors around their long axes, the scattering is equivalent to that of a single myofibril with complete statistical rotation, so all Bragg angles are sampled and there is no need to rotate the muscle sample in the X-ray beam, as is needed for conventional crystallography measurements. It is also important to note that different reflections at the same radial distance from the center in diffraction (reciprocal) space will be superimposed, so what we are calling the 1,0 reflection is actually the sum of the *h* = 1, *k* = 0; *h* = 0, *k* = 1; and *h* = 1, *k*= −1 reflection intensities.

The asymmetric crystallographic unit cell contains the equivalent of one thick filament and two thin filaments, as shown in Figure 2. We evaluated the Fourier transform of this object only at the reciprocal hexagonal lattice positions in 2D and then converted the predicted structure factors to the predicted intensities. The computational approach is similar to others published previously [24,57]. We also compared this method to several other methods of calculating the transform to confirm that they all delivered equivalent results (see Supplemental Information, Figures S1 and S2).

For each equatorial reflection with the index (h,k), the Fourier transform of the unit cell can be computed by:

(1) $$F_{hk}=F_{hk}^{thick}+F_{hk}^{thin_{1}}\cos\theta_{1}^{hk}+F_{hk}^{thin_{2}}\cos\theta_{2}^{hk},$$

where $F_{hk}^{thick}$ and $F_{hk}^{thin}$ are Fourier transforms of the projections of thick and thin filaments, and the cosine terms account for the phase difference of the two thin filaments in the unit cell with respect to the thick filament at the origin, in which $\theta_{1}^{hk}$ and $\theta_{2}^{hk}$ are defined by:

(2) $$\theta_{1}^{hk}=2\pi \left(\frac{k}{3}+\frac{2h}{3}\right),\;\theta_{2}^{hk}=2\pi \left(\frac{2k}{3}+\frac{h}{3}\right)$$

for given electron densities for thick and thin filaments $\rho_{thick}\left(r\right)$ and $\rho_{thin_{i}}\left(r\right)\;\left(i=1,\;2\right)$, $F_{hk}^{thick}$ and $F_{hk}^{thin_{i}}$ can be computed through:

(3) $$F_{hk}^{thick}=F^{thick}\left(hb_{1}+kb_{2}\right)=\int_{U}^{}\rho_{thick}\left(r\right)\exp\left(2\pi ir\cdot \left(hb_{1}+kb_{2}\right)\right)dr,$$

and

(4) $$F_{hk}^{thin_{i}}=F^{thin_{i}}\left(hb_{1}+kb_{2}\right)=\int_{U}^{}\rho_{thin_{i}}\left(r\right)\exp\left(2\pi ir\cdot \left(hb_{1}+kb_{2}\right)\right)dr,\;i=1,2$$

where U is the unit cell, and $b_{1}$ and $b_{2}$ are reciprocal lattice primitive vectors (of magnitude proportional to 1/***a***_1_ and 1*/**a**_2_*, respectively (see Figure 2), associated with the unit cell (see Supplemental Material). The disorder effects (random isotropic displacements) can be taken into account by multiplying F(S) by a “temperature” factor term [24,58].

(5) $$D_{\alpha }=exp\left(-2\pi^{2}{\left|S\right|}^{2}\Delta_{\alpha }^{2}\right),$$

where $\Delta_{\alpha }$ (α=A,M) corresponds to the root mean square isotropic displacement of thick and thin filaments, respectively, and |S| is the distance from the origin in reciprocal space.

The intensity, $I_{hk},$ is proportional to $|F_{hk}$|^2^. To compare the calculated and experimental intensities, the calculated intensities need to be divided by the Lorentz factor (L), which is equivalent to the radius in reciprocal space to a particular reflection defined by the Miller indices (h, k) [1,24]:

(6) $$L=\sqrt{h^{2}+k^{2}+hk}$$

Finally, because of the rotational averaging, the observed intensities for a given (h, k) are the sum of all reflections that have the same value of L. To evaluate the goodness of fit for the predicted intensities to the experimental data, we compute the crystallographic *R* factor [3].

(7) $$R=\frac{\sum_{i=1}{\left(I_{i}^{o}-I_{i}^{p}\right)}^{2}}{\sum_{i=1}{\left(I_{i}^{o}\right)}^{2}},$$

where $I_{i}^{o}$ are the experimentally measured mean intensities of the i-th reflection$,\;\mathrm{and}\;I_{i}^{p}$ is the computed predicted intensity for the i-th reflection. The best model parameters are obtained by minimizing the *R*-factor. It should be noted that using a different formulation of the *R*-factor taking into account the errors in the data (a “weighted *R* factor”) [23] did not change any conclusions.

### 4.4. Experimental Equatorial X-ray Diffraction Intensity Measurements

For testing the methodology developed in this paper, we chose to fit an experimental X-ray diffraction dataset obtained from a re-analysis of the X-ray diffraction patterns reported in Gong et al., 2022 [59]. Briefly, small-angle X-ray diffraction patterns were collected from resting and isometrically contracting membrane intact rat extensor digitorum longus (EDL) muscle. X-ray diffraction patterns were analyzed using the MuscleX software suite v.1.21.0 developed by BioCAT [60]. X-ray patterns were selected that showed eight observable diffraction peaks on the equator. The equatorial reflections were analyzed using the equator routine in the MuscleX suite as described previously [14], assuming a Gaussian model for the eight observable equatorial diffraction peaks (*1,0; 1,1; 2,0; 2,1; 3,0; 2,2, 3,1; 4,0*), plus an additional peak for the Z-line reflection [61]. Additional methodological details are provided in the Supplementary Materials (references [62,63,64,65,66,67,68,69,70,71,72,73,74,75] are cited in Supplementary Materials).

## Figures and Tables

**Table 1:** Average values of sarcomere geometry obtained from X-ray diffraction experiments for rat EDL muscle.

Description	Parameter	Value
Sarcomere length	*SL* (μm)	2.862
Interfilament spacing	*d*_10_ (nm)	34.21
Distance between actin and myosin	*d_AM_* (nm)	22.81

**Table 2:** Model parameters for fast (EDL) rat skeletal muscle.

Description	Parameter	Value
Myosin–actin binding rate	$k_{+A}^{}$ (s^−1^)	100
ADP release rate	$k_{+D}^{}$ (s^−1^)	300
Parked-state amplitude	$k_{PS}^{max}$ (s^−1^)	220
Parked-state baseline rate	$k_{PS}^{0}$ (s^−1^)	40

**Table 3:** Experimental and simulated steady-state tensions for rat EDL muscles during fixed-end tetani.

	Experiment (kPa)	MUSICO (kPa)
Mean isometric tension (avg. over 6 trials)	66.3 ± 13.7	66.15

**Table 4:** Predicted Fractions of Myosin Heads in the Parked State, Unbound States and Bound to Actin.

	Relaxed	Contracted
Fraction of bound crossbridges %	1.95	22.93
Fraction of unbound crossbridges %	17.67	42.67
Fraction of crossbridges in PS %	80.38	34.40

**Table 5:** Intensities of equatorial reflections of relaxed and contracting EDL muscle.

	Reflection Phase	(1.0) +	(1.1) +	(2.0) −	(2.1) −	(3.0) +	(2.2) +	(3.1) −	(4.0) −	R-Factor
Experimental Resting	Mean	100.00	36.34	15.72	6.24	6.73	0.31	2.36	1.24	
	Error		3.49	1.43	1.07	1.13	0.23	0.46	0.27	
Simulation $\Delta_{M}$ = 2.58 nm $\Delta_{A\;}$= 2.15 nm	Mean	100.00	34.05	18.73	9.13	1.77	0.35	1.17	0.22	0.0043
	Error		0.46	0.05	0.08	0.02	0.01	0.01	0.00	
Experimental Contracting	Mean	100.00	68.75	20.49	12.96	2.51	3.78	0.60	1.36	
	Error		3.72	5.27	1.00	2.25	0.38	9.75	3.72	
Simulation $\Delta_{M}\;$ = 2.58 nm $\Delta_{A}\;$ = 1.72 nm	Mean	100.00	70.12	20.34	15.69	2.96	0.81	2.49	0.41	0.00082
	Error		3.31	0.38	0.50	0.18	0.08	0.06	0.01	

## Data Availability

All datasets generated for this study are included in this article. The raw data are available from the corresponding author (Thomas Irving: irving@iit.edu) upon reasonable request.

## Supplementary Materials

The following supporting information can be downloaded at https://www.mdpi.com/article/10.3390/ijms24108474/s1.
