# Supplementary material for: Using Multiscale Simulations as a Tool to Interpret Equatorial X-ray Fiber Diffraction Patterns from Skeletal Muscle
Source: Int J Mol Sci. 2023 May 9;24(10):8474. doi: 10.3390/ijms24108474 (PMC10218096; doi:10.3390/ijms24108474)
Supplement: Supplementary file 1 [file ijms-24-08474-s001.zip › ijms-2349723-supplementary.pdf]

Supplementary Material

Basic X-ray diffraction theory

According to basic X-ray diffraction theory, for a given electron density distribution $\rho(\boldsymbol{r})$, the corresponding intensity $I(\boldsymbol{S})$ at $\boldsymbol{S}$ is proportional to $|F(\boldsymbol{S})|^2$, where $F(\boldsymbol{S})$ is the Fourier transform of $\rho(\boldsymbol{r})$, given by

$$F(\boldsymbol{S}) = \int_{\mathbb{R}^2} \rho(\boldsymbol{r}) e^{2\pi i \boldsymbol{S}\cdot\boldsymbol{r}} d\boldsymbol{r}.$$

If $\rho(\boldsymbol{r})$ is a periodic function satisfying $\rho(\boldsymbol{r} + \boldsymbol{R}_l) = \rho(\boldsymbol{r})$, where $\boldsymbol{R}_l = l_1\boldsymbol{a}_1 + l_2\,\boldsymbol{a}_2$, $\mathbf{a}_1$ and $\mathbf{a}_2$ are the primitive vectors of the unit cell represented by Cartesian coordinates, $\mathbf{a}_1 = (a_{11}, a_{12})$, $\mathbf{a}_2 = (a_{21}, a_{22})$, and the integer l_i (i=1, 2) are indexes. Since the periodic density function $\rho(\boldsymbol{r})$ can be represented by

$$\rho(\mathbf{r}) = (\rho_0 * \delta_\Gamma)(\mathbf{r}),$$

where $\rho_0(\mathbf{r})$ in the density function of the unit cell U, and $\delta_\Gamma = \sum_{\boldsymbol{l}} \delta(\mathbf{r} - \mathbf{R}_{\boldsymbol{l}})$. According to the property of the Fourier transform, we have

$$F(\mathbf{S}) = \left(\int_U \rho_0(\mathbf{r}) e^{i2\pi\mathbf{S}\cdot\mathbf{r}} \mathrm{d}\mathbf{r}\right)\left(\sum_{\boldsymbol{l}} e^{i2\pi\mathbf{S}\cdot\mathbf{R}_l}\right)$$

$$= F_0(\mathbf{S}) \sum_{l_1=0}^{N_1} \exp(2\pi i\mathbf{a}_1 \cdot \boldsymbol{S}) \sum_{l_2=0}^{N_2} \exp(2\pi i\mathbf{a}_2 \cdot \boldsymbol{S})$$

where U is the unit cell and $F_0(\mathbf{S})$ is the Fourier transform in the unit cell. When N_1 and N_2 are very large, the summation $\sum_{l_i=0}^{N_i} \exp(2\pi i\mathbf{a}_i \cdot \mathbf{S})$ are almost equal to zero unless $\mathbf{a}_i \cdot \mathbf{S}$ is an integer. So $F(\mathbf{S})$ is non-zero unless

$$\mathbf{S} = h\mathbf{b}_1 + k\mathbf{b}_2,$$

where $\mathbf{b}_1 = (b_{11}, b_{12})$ and $\mathbf{b}_2 = (b_{21}, b_{22})$ are reciprocal primitive vectors satisfying $\mathbf{a}_i \cdot \mathbf{b}_j = \delta_{ij}$. We denote $F_0(h,k) = F_0(h\mathbf{b}_1 + k\mathbf{b}_2)$ $I(h,k) = I(h\mathbf{b}_1 + k\mathbf{b}_2)$. The above analysis indicates that

$$I(h,k) \propto |F_0(h,k)|^2,$$

where

$$F_0(h,k) \triangleq F_0(h\mathbf{b}_1 + k\mathbf{b}_2) = V \int_0^1 \int_0^1 \rho_0(x\mathbf{a}_1 + y\mathbf{a}_2)\exp(2\pi i(hx + ky))\mathrm{d}x\mathrm{d}y,$$

where V is the volume of the unit cell. $I(h,k)$ may require various corrections depending on geometry of the experiment and kinds of disorder present.

Other Computational Approaches for Equatorial Intensity Calculations

Besides the computational approach described in the main text, there were two other computational approaches we tried. In the second approach, we first assemble the electron density of one thick filament and two thin filaments together. There are two equivalent ways to assemble the electron densities, as shown in 1. We can view a motif as the smallest repeated unit in the periodic array, located at each lattice position, which is a analogy to an atom in crystallography. Mathematically, we can show this approach is equivalent to the first approach. To illustrate this point, let's consider a simple case in which the density distribution $\rho(\mathbf{x})$ can be written as $\rho(\mathbf{x}) = \rho_1(\mathbf{x}) + \rho_2(\mathbf{x})$. By a direct calculation shows that

$$
\begin{aligned}
F(\mathbf{S}) &= \int \rho(\mathbf{x})\exp(2\pi i\mathbf{S}\cdot\mathbf{x})\mathrm{d}\mathbf{x} \\
&= \int (\rho_1(\mathbf{x}) + \rho_2(\mathbf{x}))\exp(2\pi i\mathbf{S}\cdot\mathbf{x})\mathrm{d}\mathbf{x} \\
&= \int \rho_1(\mathbf{x})\exp(2\pi i\mathbf{S}\cdot\mathbf{x})\mathrm{d}\mathbf{x} + \int \rho_2(\mathbf{x})\exp(2\pi i\mathbf{S}\cdot\mathbf{x})\mathrm{d}\mathbf{x} \\
&= \int \rho_1(\mathbf{x}_1 + \mathbf{y})\exp(2\pi i\mathbf{S}\cdot(\mathbf{x}_1 + \mathbf{y}))\mathrm{d}\mathbf{y} + \int \rho_2(\mathbf{x}_2 + \mathbf{y})\exp(2\pi i\mathbf{S}\cdot(\mathbf{x}_2 + \mathbf{y}))\mathrm{d}\mathbf{y} \\
&= \exp(2\pi i\mathbf{S}\cdot\mathbf{x}_1)\,\mathrm{F}_1(\mathrm{S}) + \exp(2\pi i\mathbf{S}\cdot\mathbf{x}_2)\,\mathrm{F}_2(S),
\end{aligned}
$$

where $F_i(S) = \int \tilde{\rho}_{\mathrm{i}}(\mathbf{y})\exp(2\pi i\mathbf{S}\cdot\mathbf{y})\,\mathrm{d}\mathbf{y}$ with $\tilde{\rho}_{\mathrm{i}}(\mathbf{y}) = \rho_{\mathrm{i}}(\mathbf{y} + \mathbf{x}_1)$ and $\mathbf{x}_{\mathrm{i}}$ is the center of the density ρ_i. Here $\exp(2\pi i\mathbf{S}\cdot\mathbf{x}_{\mathrm{i}})$ is the factor for the phase differerence of ρ_i with ρ. In other word, we can either first add the densities together to compute its Fourier transform or first comptute the Fourier transform and add the results together.

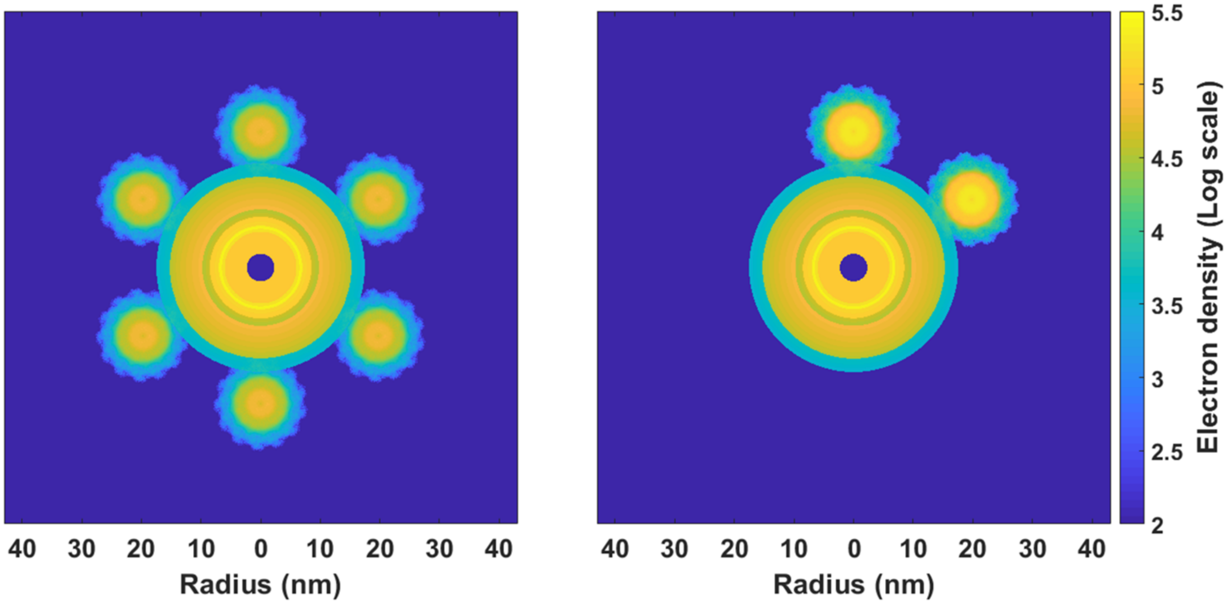

Figure S1: Two representations of the electron density in one repeating motif comprising one thick filament and two thin filaments. Part A shows the hexagonal symmetry explicitly. Because each thin filament is shared between 3 thick filaments, the density in each thin filament is reduced to 1/3 of a thin filament density shown in part B. Both representations give equivalent results.

In the third approach, we assemble a large array with N × N unit cells directly, and compute the Fourier transform of the full array. An advantage of this approach is that we can account for disorder effects explicitly, by specifying displacements of each filament from their perfect lattice positions. This approach also enable us to study other non-periodic cases, e.g. different unit cells having different densities, and cases with a finite number of unit cells. Fig. 2 shows the electron densities for arrays with 5 x 5 unit cells and 21 x 21 unit cells respectively. In both cases, it is assumed that all filaments are located in their perfect lattice positions. A disadvantage of this approach is the increased computation costs relative to other two techniques as well as a potential loss in precision, and the presence of edge effects, due to the finite lattice size. However, the calculated intensities from the 1 X 1 unit cell, direct fourier transform, calculation and 21 X 21 unit cell array calculation showed good agreement, especially for the low-orderintensities with divergence at higher diffraction orders. For example, there is a discrepancy of approximately 10% for the (4,0) intensity between the intensities obtained from the 1 X 1 unit cell and the 21*21 unit cells.

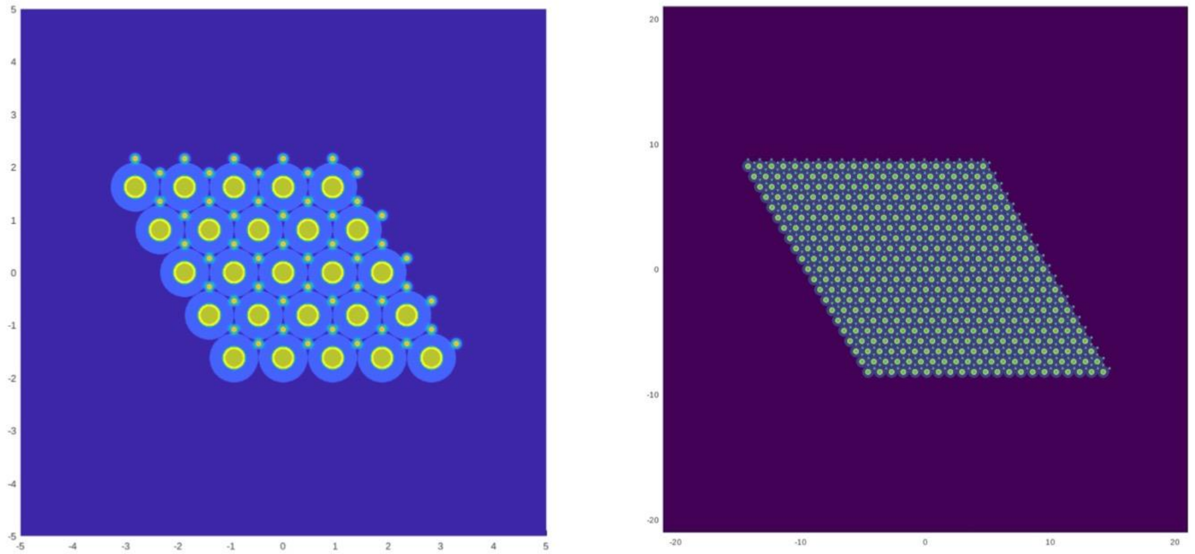

Figure S2: Electron densities in large arrays with multiple unit cell (left: 5 × 5 unit cells; right: 21 × 21 unit cells)

Resolution of the electron density distributions.

The electron density data was originally created with a resolution of 1 pixel per 0.01 nm, resulting in 8601 X 8601 pixels for each filament. However, computing the Fourier transform using this original resolution incurs a large computational cost. To reduce the computational burden, we tried mapping the electron density onto a coarser mesh. Numerical tests have shown that a resolution of 801 X 801 pixels was sufficient, with each pixel corresponding to 0.11 nm. We compared the X-ray intensities obtained from the original resolution with those obtained from the coarse-grained density and found that the difference between normalized intensities from the high resolution and the low-resolution density distributions was small (less than 0.05%).

Determining best fit parameters for the thick filament backbone model

The fittable parameters in the thick filament backbone model are the radial center of mass of the PS heads (denoted by x) and the thickness of the PS region (denoted by y) (Figure 4 in the main text). We determine these two parameters using the relaxed muscle diffraction data by brute-force parameter fitting with the fixed parked state ratio, provided by MUSICO simulations (PS ratio = 0.8 for EDL muscle). We varied the radial center of mass of the PS heads from 11.5 nm to 14 nm, and the thickness of the PS region from 4 nm to 6.5 nm. Table S1 shows the best fits, as judged by the values that yielded the smallest R-factor for EDL muscle. On this basis, we chose the radial center of mass of the PS heads to be 12.5 nm and the thickness of PS region to be 5.5 nm. We also show the dependence of the R-factor for fitting the EDL resting diffraction data with respect to the x and y parameters in Fig S4, indicating that the parameters that we picked are optimal.

Table S1 Best Fit parameters for EDL resting data (fixed Δ_M= 2.58 nm, Δ_A= 2.15 nm)

X	y	(1, 0)	(1,1)	(2,0)	(2,1)	(3,0)	(2,2)	((3,1)	(4, 0)	R-factor
12.5	6	100	34.58	17.68	9.40	1.57	0.30	1.24	0.21	0.0039
12.5	6.5	100	34.89	17.62	9.82	1.47	0.29	1.23	0.19	0.0042
**12.5**	**5.5**	**100**	**33.94**	**18.67**	**9.16**	**1.79**	**0.35**	**1.20**	**0.22**	**0.0043**
13	5.5	100	39.87	19.86	8.57	2.34	0.34	1.66	0.31	0.0048
13	6	100	40.29	18.92	9.01	2.06	0.30	1.66	0.28	0.0049
13	6.5	100	40.60	18.92	9.47	1.95	0.31	1.60	0.25	0.0054
12.5	5	100	33.06	20.54	8.89	2.17	0.45	1.11	0.23	0.0055
13	5	100	39.25	21.74	8.17	2.83	0.42	1.63	0.34	0.0056
12	6.5	100	31.20	16.00	10.25	1.04	0.26	0.99	0.14	0.0067
12	6	100	30.79	16.12	9.94	1.12	0.27	0.97	0.16	0.0068

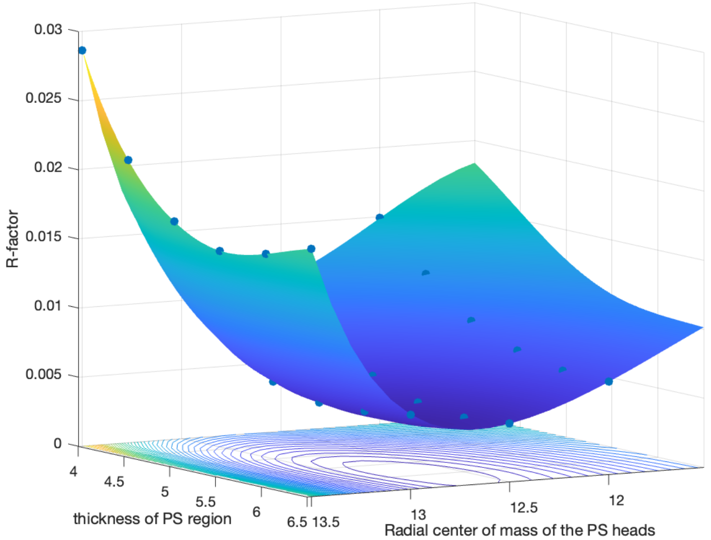

Figure S4 R-factor with respect to x (radius center of mass of the PS head) and y (thickness of PS region) for EDL resting data **(**Δ_M= 2.58 nm Δ_A= 2.15 nm**)**.

MUSICO simulation parameters

Table S2. MUSICO parameters for all simulations of isometric contractions in EDL rat muscles.

Description	Parameter	Value
**Crossbridge Cycle**		
Myosin-actin binding rate	k+A0	100 s^{-1}
Myosin-actin detachment rate[a]	k−Ao	40 s^{-1}
Working stroke [62-65]	d	10.5 nm
Second working stroke [62-65]	δ	1 nm

Myosin reverse stroke cap rate[b]	k−Picap	33 s^{-1}
ADP release rate	k+D0	300 s^{-1}
ATP binding and myosin detachment rate constant[a]	k+T	10^6 s^{-1}
Hydrolysis forward rate[a]	k+H	150 s^{-1}
Hydrolysis backward rate[a]	k−H	15 s^{-1}
Crossbridge stiffness [62-65]	κ	1.3 pN/nm
kBT at 25 °C	kBT	4.116 pN·nm
**Parked State**		
Transition rate constant to "parked state"[c]	k−PS	200 s^{-1}
Baseline rate constant	kPS0	40 s^{-1}
Amplitude	kPSmax	220 s^{-1}
Calcium Hill function slope[c]	b	5
Half activation point of the Hill function[c]	Ca50	1 µM
**Calcium Kinetics**		
Calcium binding to TnC equilib. rate constant [66,67]	K~Ca	10^6 M^{-1}
Calcium binding rate constant to TnC [66,67]	k~Ca	$7.54 \cdot 10^7$ $M^{-1} \cdot s^{-1}$
Calcium dissociation rate constant from TnC [68-70]	k−Ca	75.4 s^{-1}
TnI-actin equilibrium rate const. at high Ca^{2+}	λ	10
TnI-actin backward rate const.	λ−	375 s^{-1}
TnI-actin-Ca cooperativity coefficient [65,67]	εo	0.01
**CFC**		
Tropomyosin pinning angle [71]	ϕ−	-25°
Myosin Tm angular displacement [71]	ϕ+	10°
Angular standard deviation of free CFC [72,73]	σ0	29.7°
Persistence length of Tm-Tn confined chain [73]	$1/\xi$	50 nm
**Sarcomere**		
Sarcomere Length	SL	2.2 µm
Reference length of actin filament [74,75]	Lao	1.1 µm
Interfilament spacing at SL=2.2 µm [76]	d10	33.8 nm
Thin filament elastic modulus [77,78]	AEa	65 nN
Thick filament elastic modulus [77]	AEm	132 nN

[a] Based on mouse and human α-myosin values in [79,80], with corrections for temperature and ionic strength as documented in [81].

[b] The power stroke rates (k+Pi and k−Pi) are expected to be slower in β-isoforms for ~5 fold and this is achieved by reducing adapting Gstroke and decreasing k−Picap by factor ~3; For the same isoform the power stroke rate increase from humans to rats and to mice is accomplished by increase in k−Picap.

[c] Assumed.
